# Progress of Cholera in Chicago

**Published:** 1854-07

**Authors:** 


### EDITORIAL.

*Progress of Cholera in Chicago.*

In the June Number of the Journal we gave a detailed account of the prevalence of Cholera, &c., up to the 28th of that month. In the present number will be found a digest of what has appeared, concerning the nature of the disease, in foreign Journals; and we have thought it not unprofitable to continue our sketch of its progress here in connection with such notes of local circumstances and atmospheric changes as have fallen under our observation. At the time of our last issue (28th of June) the prevalence of Cholera in the City had not materially increased during the preceding week, and without any positive data, we than expressed the opinion that the number of fatal cases during the month had averaged 4 or 5 per day. At the close of the month the report of the City Sexton showed the whole number of burials during the month of June to have been 333, of which 142 were reported to have resulted from deaths by Cholera. Still the subject received no public attention from the Board of Health and the daily city papers continued to report the health of the city as very good; some of them positively denying that any such disease as Cholera existed among us. But the FELL DESTROYER paid no heed to newspaper reports, and the disease continued slowly increasing in its prevalence from the 28th of June to the 4th of July. From the 4th of July to the 8th, the disease increased with remarkable rapidity; and instead of being confined within particular blocks or sections of the city, as in the earlier part of the season, it marked its victims in every street, and among all classes of citizens. On Wabash and Michigan Avenues, streets in other seasons almost exempt from Cholera, signals of mourning were to be seen on almost every block. So rapid was the increase of the disease that during the first 8 days of the month the City Sexton reported the whole number of deaths to be 242, and more than half of these occurred during the last three days of the eight. During all these days the weather had been intensely hot and the atmosphere very damp, though very little rain fell during the whole period. On the 6th, 7th and 8th especially, the air was so nearly saturated with moisture and the temperature so high, that persons in the best of health found it very oppressive. — It was during these days that the disease assumed its most malignant form, and appeared in all parts of the city and among all classes. It evidently reached its crisis or greatest prevalence on the 8th. On the 9th there was an evident diminution of attacks, and from that time to the 14th the daily mortality of the city remained nearly stationary. The whole number of deaths each day were:

| | | | |
|---|---|---|---|
| on the 9th | 38 | 12th | 38 |
| 10th | 37 | 13th | 35 |
| 11th | 44 | 14th | 36 |

From the 14th to the 17th the mortality decreased as follows, viz: On the 15th, deaths - - - - - - - 28

| | | | |
|---|---|---|---|
| 16th | 16 | 17th | 22 |

For the remainder of the month the whole number of deaths have been reported as follows, viz:

| | | | |
|---|---|---|---|
| on the 18th | 28 | 25th | 29 |
| 19th | 30 | 26th | 23 |
| 20th | 28 | 27 | 30 |
| 21 | 20 | 28 | 33 |
| 22 | 32 | 29 | 36 |
| 23 | 23 | 30 | 29 |
| 24 | 24 | 31 | 37 |

In comparing the outward or appreciable state of the atmosphere with the foregoing, we shall find some striking co-incidents, if they do not bear the relation of cause and effect.

For instance, from the 3d to the 8th of July the weather was intensely hot. On the 4th there was a slight sprinkling of rain with lightning, and the atmosphere from that time to the 8th was highly saturated with moisture; so much so, that porous paper nailed to the dry wall of my office became swollen with moisture, and the proper tests showed a high degree of saturation. During most of the time also, the wind was from the south or south-west, coming over the most extensive low level prairies. During the 6th, 7th and 8th, the attacks of Cholera were not only numerous but they were extremely malignant.

The vital forces seemed to be overcome as if by an overwhelming shock. Re-action was extremely difficult to establish, and there was no tendency to secoudary inflammation or dysentery.

About middle of the afternoon of the 8th, the wind suddenly changed to the North and North-East and blew from the Lake so cool as to produce decided feelings of chilliness, and just at night there came a violent and copious thunder shower.

From the 9th to the 17th the weather was only moderately warm; the winds were variable, and during the latter part of the time especially the air was less saturated with moisture.

On the 18th and 19th it again became intensely hot, the mercury rising, each day, to near 100° at 12 M. in the shade.

On the 20th at noon it was 4° lower, and in the afternoon of that day the wind again shifted to the North-East, blew cool from the lake, and was followed by thunder showers. It remained comparatively cool, with winds from the north, and some rain for several days. — From the 26th to the present time (31st) the sky has been mostly clear, the winds from the South and South-West, the temperature ranging from 90° to 100° at mid-day in the shade, and still the degree of atmosphere moisture above the ordinary range.

The sudden change in the weather which we have noted as occurring on the afternoon of the 8th, was followed within 24 hours by a very marked change in the *character* of the prevailing epidemic. The number of those attacked with Cholera or Cholera symptoms, was not materially diminished during the next three or four days, but the specific character of the disease was evidently changed. Those cases, which were seen early, yielded much more readily to treatment.

And when the active Cholera discharges were arrested, inflammatory action in the mucous membranes was more readily set up, especially if heating and stimulant mixtures had been taken in commencement. In many of the cases symptoms of dysentery were mixed with those of Cholera almost from the beginning of the attack, accompanied by heat of skin and a firm pulse. These changes in the aspect of the prevailing disease were so plain, and followed so quickly the sudden change in the atmospheric temperature which took place on the afternoon of the 8th, that I feel no hesitation in regarding them as necessarily connected with each other.

From the period of which we are now speaking to the present time, the prevalence of dysentery in the city has increased, until the number of cases now greatly exceed those of Cholera. The latter disease evidently increased during the extreme hot days from the 17th to the 20th, remained nearly stationery until the 26th, since which time it has slightly increased.—The only inference which we can draw from a comparison of the prevalence of Cholera with the atmosphere changes, is that high heat, accompanied by South and South-West winds and unusual moisture, have uniformly, this season, increased the disease. By atmospheric moisture we mean that which is held in solution in the air, and is derived from evaporation from the surface of the soil, without reference to falling rain. The latter, especially when copious, often purifies the atmosphere and washes noxious materials from the earth's surface, while the former is often loaded with noxious matter in a form most favorable for entrance into the system through the pulmonary and cutaneous surfaces. The summer of 1853 was characterized by an unusually dry atmosphere throughout the whole North-West. This season, so far as my examinations go, we have had in this City a degree of atmospheric moisture much above the average, ever since the 20th of April.

It will be seen from the figures we have given, that the whole number of deaths during the month of June was 333; and during the month of July 948. The exact proportion of these resulting from Cholera alone, it is impossible to ascertain. It is true, that since the 8th of July the Board of Health have issued a report daily, purporting to give both the whole number of deaths and the number from Cholera. But in reference to the latter, their statements are entirely unreliable, they having enforced no adequate law for ascertaining the causes of death in any instance. A very slight examination will show the truth of this assertion.—For instance in most of their daily reports, in from one quarter to one third of the whole number given, the causes of death are stated to be *unknown*, and yet in the footing up these *unknown* are always placed in the list of "other diseases". By such a process they have kept the number of cholera cases comparatively low, but have at the same time unwittingly swelled the number of deaths from ordinary diseases to such an extent, as to indicate a far worse state of public health than though they had doubled the number of deaths from Cholera or any other temporary or special Epidemic. — Thus on the 17th inst., the Board reported the whole number of deaths to be 23, of which only *three* were from Cholera, leaving 19 deaths in one day from *other* and ordinary diseases. Now the usual mortality from ordinary diseases, without the addition of any special epidemic at this season of the year, should not exceed an average of four or five per day. And if the number was actually increased to 19 per day, it would indicate a sanitary condition far worse than though three times that number were dying daily from some fatal though temporary epidemic.

But if we examine the details of the report the fallacy of the general statement will be obvious at once.—The cases of death are stated as follows, viz:

| | | | |
|---|---|---|---|
| Of Typhoid Fever . | 2 | Marasmus . . | 1 |
| Bilious Fever . | 2 | Diseases of Infancy | 1 |
| Erysipelas . . | 1 | Diarrhoea . . | 3 |
| Teething . . | 3 | Cholera . . . | 3 |
| Unknown . . . . . . . . | | | 7 |

Here very nearly one third of the whole number are confessedly unknown, and what is still more strange, 4 of the 7 are from the Cholera Hospital which is directly under the superintendence of the City Physician, who is also an officer of the Board of Health. According to the Board of Health Reports the whole number of deaths in the city during the month of July was 948, of which only 566 were attributed to Cholera, thus having the enormous mortality of 359 in one month from ordinary diseases. Such statistics should never be permitted to emanate from any official source. To say that the deaths during the month from all ordinary diseases had been 175, and from Cholera alone 766, would be much nearer to the truth. As has been the case in all preceding years, the lowest class of laborers who live in the worst places and drink the most intoxicating drinks, have furnished to the Cholera the largest number of victims. The new coming emigrants have also suffered severely, adding much to the apparent mortality of the city. For a few days while the epidemic seemed to be at its crisis, it paid very little deference to either class, sex, or location. Indeed there was one section of the city which in all the previous cholera epidemics had been almost exempt, which now suffered for a few days severely. We allude to Wabash and Michigan Avenues, lying paralell with the Lake Shore in the South-Division.

These streets had been in all former years highly salubrious. But since the last prevalence of cholera in 1852, the whole length of the Lake Shore in that Division of the city has been isolated from the body of the Lake by the Illinois Central R. R Track, and its protection. Whether this partial stagnation of the water on the shore, together with the accumulation of manure and other matters which have been deposited there, have already deteriorated the healthiness of the neighboring streets, is a question of much interest and importance.

One thing in reference to the Cholera, we have watched this season, with much care and interest. We allude to its connection with Dysentery. As we have already said the latter disease began to show itself soon after the sudden and marked atmospheric change, which took place on the evening of the 8th July. At first, we noticed it only in cases which commenced with vomiting and diarrhœa, or decided cholera symptoms; the discharges after a few hours becoming small, tinged with blood, or mixed with mucus and shreds of coagulable lymph, and accompanied by cutting pains in the abdomen with slight fever. These symptoms were almost uniformly noticed, in those cases where the patient had endeavored to check the first indications of intestinal disturbance by the use of Brandy, Brandy and Cayenne Pepper, or the various heating Cholera specifics and preventatives with which the community is always well supplied. And many were thus made sick, who would not, otherwise, have suffered any material inconvenience.

In a few days, however, the attacks of Dysentery became numerous, without being preceded by any cholera symptoms, or the use of any injudicious remedies. The attacks generally began with severe paroxysmal pains in the abdomen, frequently following the track of the Colon; frequent desire to evacuate the bowels, the discharges at first being simply thin feces, sometimes of a whitish color like soap-suds, sometimes redish-brown, and at other times almost exactly resembling *blue-clay*, rendered semi-fluid by intermixture with water. At the same time, the tongue is covered with a white or yellowish-white coat, the pulse rather sharp and quick, but not full, and the skin moderately hot and dry.

If the case is not interfered with, in the course of 24 hours the discharges become more frequent with tenesmus, and in most cases they are very small in quantity, consisting of a little thin mucus and pieces of coagulable matter, more or less mixed with blood. At the same time the tongue and skin become more dry, the pulse quicker, and the patient more restless. In some of the cases we have seen, the tenesmus has been very great, creating an inclination to be on the stool almost constantly, with scarcely any discharge whatever. Only a small proportion of the attacks have been commenced with a chill, or have shown a distinct tendency to periodicity; though in some cases both the chill and the periodicity have been well marked. In all the former seasons, from 1849 to 1852, we have noticed more or less dysentery in connection with the prevalence of Cholera. But the type of fever accompanying it was essentially asthenic or typhoid, and the discharges were more copious, often consisting of a serous fluid mixed with blood, and voided without extreme pain or restlessness. This season, however, we have scarcely met with a single instance of this kind; the fever accompanying the attacks now being pretty uniformly *irritative* in its character, closely resembling the hot stage of a *mild remittent*, and the discharges indicating a more decided inflammatory condition of the mucous membrane of the rectum and colon.

It is evident that the Dysentery, now prevalent, is accompanied by a more active grade of inflammatory action in the mucous membranes and more sthenic grade of fever than that which accompanied the Cholera in the former years we have named. There is also a much more decided tendency to periodicity, manifesting more plainly the intermixture of a malarious element. Yet most of the cases met with, have been mild and readily controlled by proper treatment. The perfect intermixture of Cholera and Dysentery, and the frequent transition from the one into the other, which has been observed since the 9th of July, give rise to some interesting pathological questions concerning the relationship of the two diseases. Are they both capable of being generated by the same causes, modified in their action only by individual peculiarities and atmospheric condition ?

If so, does not the fact militate against the idea that Cholera depends upon a *specific poison* as its essential cause ? Are there any facts in the history of diseases known to arise from specific poisons analogous to the union of Cholera with Dysentery ? We think not. No changes of atmospheric or outward conditions will convert Variola into Scarlatina, or the latter into Rubeola. And, if these distinct diseases ever occur at the same time, in the same individual, each preserves its distinctive characteristics, neither being truly merged into, or susperceded by the other. Again, can that be regarded as an *essential* cause, which is susceptible of such modification by atmospheric and other outward circumstances, as to cause widely variant symptoms and results ? If the ordinary physical circumstances and conditions are conceded to be capable of ex erting so controlling an influence, would it not be more rational to place them at once in the position of direct exciting causes, instead of retaining the purely hypothetical idea of a specific poison ?

These questions we have not time now to discuss, but they are well worthy of attention.

In regard to the treatment of Dysentery, it may be well to say a few words. We have almost uniformly found the disease to yield readily to a very simple form of treatment, consisting of Calomel and Opium, in the proportion of three or four grains of the first, to one and a half or two grains of the last, given every 2, 3, or 4 hours, until from three to six doses have been taken. This generally subdues the pain and quiets the bowels.

If they remain quiet for 12 or 18 hours, give a table spoonful of the following mixture every 2 hours until it procures one or two free fecal evacuations, viz :

℞ Castor Oil, - ℥j.

Oil Turpentine, - ʒss.

Pulv. G. Arabac, } aa ʒiij.

Rub together and add
Mint Water, - ℥j. M.

As soon as the bowels have been freely moved, I give one of the following pills, and repeat them every two or four hours, until the pain and dysenteric discharges all cease, viz.:

Nitras Argenti, - grs. vij.
Pulv. Opii., - - - grs. xx.
Mix—divide into 20 pills.

In the early stage, it is sometimes necessary to use Starch and Laudanum enemas to aid in relieving the tenesmus. Sometimes instead of the Nitrate of Silver and Opium, I use in the second stage of the disease an Emulsion of Oil of Turpentine and Laudanum, with the best effects. But at no former period of my practice have I found Nitrate of Silver, in combination with Opium, as beneficial in Dysentery, as during the present summer. If the case presents any distinct evidence of Periodicity I find the Quinine in antiperiodic doses promptly beneficial. D.

---

*Indiana Medical Journal.*

We have received the first number of this new auxiliary to our periodical literature. We predict for it a successful career, should future numbers equal in merit that already received.

There are two difficulties to be met in conducting such an enterprise. The first relates to its *pecuniary* support. We are satisfied from our experience in Journalism, that the only safe course is to require payment invariably in advance, and to stop sending the Journal at the expiration of the time paid for. This course enables the publisher always to know with certainty the amount of income, and saves him the disappointment always resulting from estimates based on a large list of non-paying subscribers. It is an accommodation to subscribers themselves. Of this, we are quite certain. It is much easier to pay two or three dollars and feel no more uneasiness or anxiety on the subject, than to pay eight or ten after two or there years of torture under the lashes of a guilty conscience, or, if there is no conscience in the matter—as we sometimes fear—the vexation of continual duns.

